# A curcumin direct protein biosensor for cell‐free prototyping

**DOI:** 10.1049/enb2.12024

**Published:** 2022-08-18

**Authors:** Agata Kennedy, Guy Griffin, Paul S. Freemont, Karen M. Polizzi, Simon J. Moore

**Affiliations:** ^1^ School of Biosciences University of Kent Canterbury UK; ^2^ Centre for Synthetic Biology and Innovation South Kensington Campus London UK; ^3^ Department of Medicine South Kensington Campus London UK; ^4^ Department of Infectious Disease Section of Structural and Synthetic Biology Imperial College London London UK; ^5^ Sir Alexander Fleming Building South Kensington Campus London UK; ^6^ UK Dementia Research Institute Care Research and Technology Centre Imperial College London Hammersmith Campus London UK; ^7^ UK Innovation and Knowledge Centre for Synthetic Biology (SynbiCITE) and the London Biofoundry Imperial College Translation & Innovation Hub London UK; ^8^ Department of Life Sciences Imperial College London South Kensington Campus London UK; ^9^ Department of Chemical Engineering Imperial College London South Kensington Campus London UK

**Keywords:** biosensor, cell‐free, curcumin, fine chemical, synthetic biology

## Abstract

In synthetic biology, biosensors are routinely coupled with a gene expression system for detecting small molecules and physical signals. We reveal a fluorescent complex, based on the interaction of an *Escherichia* coli double bond reductase (*Ec*CurA), as a detection unit with its substrate curcumin—we call this a direct protein (DiPro) biosensor. Using a cell‐free synthetic biology approach, we use the *Ec*CurA DiPro biosensor to fine tune 10 reaction parameters (cofactor, substrate, and enzyme levels) for cell‐free curcumin biosynthesis, assisted through acoustic liquid handling robotics. Overall, we increase *Ec*CurA‐curcumin DiPro fluorescence within cell‐free reactions by 78‐fold. This finding adds to the growing family of protein–ligand complexes that are naturally fluorescent and potentially exploitable for a range of applications, including medical imaging to engineering high‐value chemicals.

## 1 INTRODUCTION

Fluorescence is an attractive physical property, widely exploited for sensing and quantifying small molecules for medical imaging to engineering applications [1, 2, 3]. For synthetic biology, there is a rising interest to develop fluorescent biosensors for detecting specific high‐value chemicals for heterologous production in microbial systems [4, 5]. This is important, as most chemicals require resource‐intense analytical processes (e.g. high‐performance liquid chromatography and mass spectrometry) for quantitation [6]. This type of analysis presents a bottleneck for implementing the design‐build‐test‐learn (DBTL) cycle within synthetic biology [4]. Biosensors, including riboswitches and transcription factors (TFs), are routinely coupled to the production of a fluorescence protein [7, 8, 9], RNA aptamer [10] or enzymatic reaction [11, 12] to monitor a gene expression circuit. Transcription factors (TFs) are the largest family of biosensors and use either a one‐ or two‐component cascade [13, 14, 15] to sense a range of metabolites and physical signals. Currently, about 4000 TFs are listed on the PRODORIC2® database [15]. In contrast, riboswitches are *cis* RNA elements that form secondary structures to interact with target ligands and regulate either transcription or translation. Riboswitches can detect some amino acids, metals, nucleotides and cofactors [16]. However, both riboswitches and TFs require a gene expression cascade to regulate an output device (e.g. fluorescence protein). Therefore, their sensory function is indirect, complicating the measurement workflow.

We considered that an alternative, yet generalisable, direct biosensor approach exists where the output signal transmits a direct interaction of the analyte with the biosensor. Such a mechanism bypasses the typical gene expression/maturation requirements of oxygen‐dependent fluorescent proteins in genetic circuit design [17]. In addition, it is desirable to target industrially relevant chemicals (e.g. natural products and fine chemicals) outside of the typical substrate scope of TFs (primary metabolites/physical stimuli) and riboswitches. Therefore, we reviewed the literature for potential untapped sources of novel biosensors, such as fluorescent protein–ligand complexes, whereby fluorescence provides a molecular handle to gauge ligand concentration. Examples, though few, include the nucleotide‐binding proteins, such as flavin mononucleotide (FMN)‐binding Lov proteins [18], blue‐fluorescent nicotinamide adenine dinucleotide phosphate (NADPH)‐oxidoreductase [19] and the NAD^+^/NADH binding Rex protein [1], albeit fused to a circularly permuted yellow fluorescence protein (YFP) for detection [20]. Interestingly, these interactions exploit the intrinsic fluorescence of the nucleotides FMN and NAD(P)H. An exception to this nucleotide biosensor collection is the biliverdin and bilirubin binding small ultra‐red fluorescent proteins (smURFP) [21, 22]. We speculated whether other small molecules that possess similar chemical properties as biliverdin/bilirubin (e.g. conjugation, and aromatic) and intrinsic fluorescence could provide new biosensor leads. We refer to such examples as direct protein (DiPro) biosensors. Herein, we report a curcumin DiPro biosensor, based on the intrinsic fluorescence properties of the natural product, curcumin, and a cognate binding protein. We characterise these interactions and exploit the fluorescence to create and optimise a synthetic cell‐free pathway for curcumin biosynthesis as a proof of concept.

## 2 METHODOLOGY

### 2.1 Molecular biology

Routine molecular biology was performed as described previously [23]. The TAL, PCL and MatB expression constructs were described in our previous work [24]. CUS [25] was synthesised by ThermoFisher Scientific and codon optimised for *E. coli* K12 expression with compatibility for EcoFlex cloning [23]. *Ec*CurA was PCR amplified from *E. coli* MG1655 genomic DNA using Q5 polymerase and sub‐cloned into pBP‐ORF [23]. *Ec*CurA was assembled with a strong constitutive promoter (SJM928) and N‐terminal His_6_‐tag for *E. coli* expression [23]. All oligonucleotides, plasmids and synthetic DNA sequences are listed in the supporting information. Sequencing was performed by Eurofins, UK.

### 2.2 Protein expression and purification

His_6_‐tagged recombinant TAL, PCL, CUS, MatB and *Ec*CurA were overproduced in *E. coli* BL21‐Gold (DE3) grown at 37°C, 200 rpm in 2 YT medium with 100 μg/ml ampicillin until an OD_600_ of 0.6 was reached. Cells were induced with 0.4 mM IPTG and grown overnight at 21°C at 200 rpm. Cells were collected by centrifugation at 6000 × *g*, 4°C for 20 min, then resuspended in binding buffer (20 mM Tris‐HCl pH 8 and 500 mM NaCl, 5 mM imidazole) and lysed by sonication. Cell‐lysates were clarified with centrifugation at 39,191 × *g*, 4°C for 20 min and purified by gravity flow using Ni‐nitrilotriacetic acid (NTA) agarose (Cytiva). His_6_‐tagged proteins were washed with increasing concentrations of imidazole (5, 30 and 70 mM) in 20 mM Tris‐HCl pH 8 and 500 mM NaCl, before elution at 400 mM imidazole. Purified proteins were dialysed (MWCO 10,000) for ∼16 h in 2 L of 20 mM HEPES pH 7.5 and 100 mM NaCl (Buffer A) at 4°C. All enzymes were soluble and retained activity upon storage at −80°C with 15% (v/v) glycerol. Aliquots of 20 mg/ml *Ec*CurA were stored in Buffer A without glycerol at −80°C.

### 2.3 Absorbance spectroscopy and fluorescence titrations

Absorbance and fluorescence spectra of purified *Ec*CurA and controls were measured in Buffer A using an Agilent Cary60 UV‐Vis or Cary Eclipse fluorescence spectrometer, respectively.

### 2.4 Liquid handling robotics and microplate reader fluorescence measurements

Reactions were studied in 384 or 1536 well microtiter plates (Greiner), at a volume of 10 μL or 2 μL, respectively, and prepared using an Echo® 525 acoustic liquid handling robot (LabCyte). A general Python script for robot transfer instructions is available at https://github.com/jmacdona/ODE_MCMC_tools. Liquid droplets were transferred as multiples of 25 nL to a final volume of 2–10 μL as technical triplicate replicates. Plates were sealed with Breathe‐Easy^®^ sealing membrane (Sigma) and briefly centrifuged at 1000 × *g* for 10 s. A CLARIOstar plate reader (BMG Labtech, Germany) was used for enzyme incubations and kinetic fluorescence measurements using monochromator system optics. Standard measurement settings were 30°C incubation temperature, 40 flashes per well, 10 s of 300 rpm orbital shaking prior to measurement, 0.1 s settling time, 1 min recordings for 180 cycles, 425‐10 nm excitation, 520‐20 nm emission and 1600 gain.

## 3 RESULTS

**FIGURE 1:**
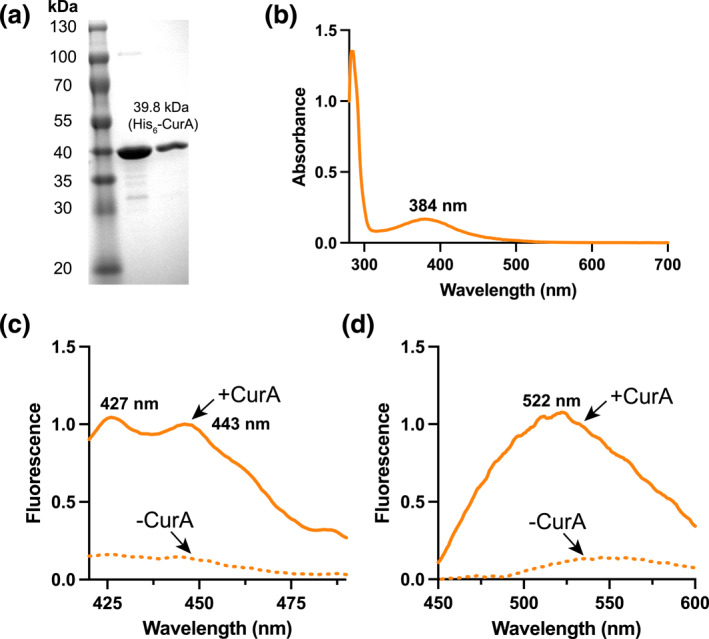
Characterisation of the *Ec*CurA DiPro biosensor. (a) SDS‐PAGE of purified His_6_‐tagged *Ec*CurA. (b) UV‐Visible absorbance of co‐purified His_6_‐tagged *Ec*CurA bound with curcumin. (c) Relative excitation and (d) emission fluorescence spectra of *Ec*CurA with and without curcumin bound (1:1 ratio—25 μM) in Buffer A at 25°C

Curcumin is a type III polyketide and a yellow pigment produced by the turmeric plant (*Curcuma longa*). From a biotechnology perspective, there is a generic interest in using microbes to make curcumin, a medicinal natural product, in order to replace land‐intense farming of turmeric [26]. Curcumin biosynthesis stems from L‐tyrosine/L‐phenylalanine metabolism. The molecule displays weak intrinsic fluorescence in aqueous solution with a quantum yield of 0.01 [27]. However, curcumin fluorescence is strongly enhanced upon non‐specific binding to extracellular curli (amyloid‐like fibres) on the surface of *E. coli* [28]—a physical property that we sought to exploit. Based on this known fluorescence property, we investigated the curcumin binding properties of the NADPH‐dependent *E. coli* curcumin reductase (*Ec*CurA—NCBI RefSeq: WP_000,531,452), as a potential soluble intracellular protein that may mirror the curcumin–curli fluorescence interaction. To begin, we constitutively expressed and purified N‐terminal His_6_‐tagged *Ec*CurA to homogeneity. A single litre of *E. coli* BL21 (DE3) Star culture in 2 YT provided approximately 20 mg of pure apoprotein with an observed molecular weight of ∼40 kDa (His_6_‐tag‐*Ec*CurA is 39.6 kDa) by denaturing gel electrophoresis (Figure 1a). Favourably, *Ec*CurA accumulated to  ∼30% of the total protein content in *E. coli* and was located entirely in the soluble fraction. Post‐purification, *Ec*CurA was highly stable, and up to 30 mg/ml and was stored in the freezer without cryoprotectants (e.g. glycerol and DMSO). In addition, we also incubated an excess of curcumin with the cell‐extract and purified the protein from this mixture (see methods). This resulted in a visual co‐purification of *Ec*CurA in complex with curcumin. Curcumin displayed an absorbance maximum at 425 nm (*ε* = 23,800 M^−1^ cm^−1^) in aqueous solution [29]. In contrast, UV‐Visible absorbance spectra of the co‐purified *Ec*CurA‐curcumin complex was blue‐shifted to 384 nm for the curcumin absorbance maxima (Figure 1b). Next, we incubated 25 μM of the apoprotein with 25 μM of curcumin. Binding of curcumin to *Ec*CurA generated strong yellow fluorescence, with broad absorption and emission spectra with maxima at 427 and 522 nm, respectively (Figure 1c). Fluorescence was relatively stable up to an hour, although curcumin degrades spontaneously in alkaline conditions [27]. Since *Ec*CurA has catalytic activity as a double bond reductase, the addition of an excess of NADPH (1 mM) led to substrate turnover, resulting in a loss of conjugation and visual colour/fluorescence. While previous studies on the *Ec*CurA and related *Vibrio vulnificus* CurA (*Vv*CurA) have established the kinetics of CurA [30, 31], both enzymes were studied at sub‐stoichiometric levels. In contrast, by incubating 25 μM of *Ec*CurA with a titration of curcumin (0–100 μM), the fluorescence signal fitted an exponential curve with a *K*_*D*_ of 8.39 μM (Figure S1–S3). In addition, the *Ec*CurA DiPro biosensor showed a reasonable limit of detection of 0.29 μM for curcumin, calculated as previously described [32].

**FIGURE 2:**
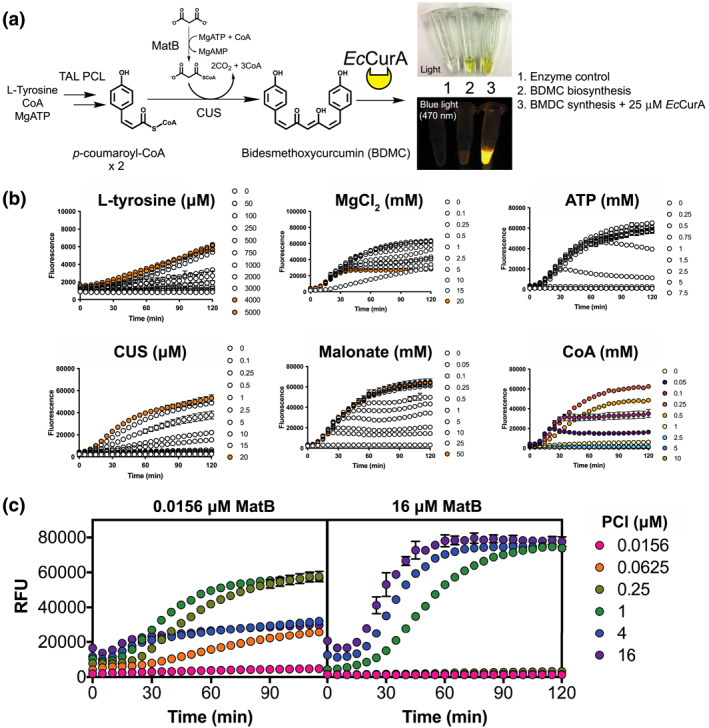
Optimisation curcumin biosynthesis with the DiPro biosensor, assisted with acoustic liquid handling robotics. (a) A synthetic pathway for detection of BDMC synthesis from malonyl‐CoA and *p*‐coumaroyl‐CoA. *Ec*CurA binds to BDMC, generating a unique fluorescent output for relative quantitation of pathway activity. Inset visual image shows visible fluorescence of cell‐free BDMC reactions, with negative controls. (b) Fine‐tuning of cofactors and substrates, and (c) enzymes to maximise BDMC fluorescence signal detection with *Ec*CurA. Unless indicated, standard reaction conditions contained 1 mM L‐tyrosine, 5 mM MgCl_2_, 10 mM malonate, 5 mM ATP, 0.25 mM CoA, 1 μM TAL, 1 μM PCL, 1 μM MatB, 25 μM CUS and 25 μM *Ec*CurA. Enzyme levels were varied in a 4‐fold dilution series from 16 to 0.0156 μM. Where two enzymes were varied, the third enzyme was kept constant at 1 μM. 10 μl reactions were performed at 30°C as a technical triplicate repeat, prepared with the liquid handling robot. The assay was repeated as two biological repeats to ensure reproducibility

Next, we sought to show the potential of the *Ec*CurA DiPro biosensor using a cell‐free synthetic biology strategy. Here, we added an excess of the *Ec*CurA DiPro biosensor in a purified multienzyme system in the absence of NADPH. Curcumin biosynthesis, like other type III polyketides, requires the precursors *p*‐coumaroyl‐CoA (and analogues) and malonyl‐CoA. We therefore created a synthetic enzyme pathway to supply these precursors and study the biosynthesis of curcumin. For this, we used the TAL, *p*‐coumaroyl‐CoA ligase (PCL) and malonyl‐CoA synthetase (MatB), as described in our previous study [24], along with a curcumin synthase (CUS) from *Oryza sativa* [25]. To establish initial reaction conditions, 1 mM of L‐tyrosine, the cofactors (ATP, malonate, CoA, and Mg^2+^) and 1 μM of purified enzymes (TAL, PCL, MatB and CUS) were incubated at 30°C to synthesise the analogue of curcumin, bisdemethoxycurcumin (BDMC). First, initial reactions accumulated a yellow visual appearance (Figure 2a). Critically, the reaction product was fluorescent only in the presence of the *Ec*CurA (25 μM) DiPro biosensor (Figure 2a) and we confirmed the biosynthesis of the product from the reaction using reverse‐phase (C‐18) high‐performance liquid chromatography (data not shown). Next, we prepared a time‐course reaction to monitor BDMC biosynthesis using the *Ec*CurA DiPro biosensor as a detector. Here, increased fluorescence, with excitation at 425–10 nm and emission at 520–20 nm, was observed, relative to the background. Importantly, if the *Ec*CurA DiPro biosensor or any of the biosynthetic enzymes or substrates were omitted, fluorescence was similar to background levels (Figure S4). Furthermore, if an aliquot of NADPH (1 μM) was spiked into the reaction, a drop in fluorescence was observed, showing that *Ec*CurA‐dependent reductase activity quenches fluorescence. These results confirmed the sensitivity and specificity of the DiPro biosensor assay for BDMC biosynthesis.

An important advantage for a cell‐free multienzyme system is the ability to rapidly test multiple parameters simultaneously. Therefore, to study enzymatic synthesis of curcumin in cell‐free reactions, we used the *Ec*CurA DiPro biosensor to monitor multiple reactions in parallel using real‐time fluorescence measurements. Using the same conditions as described earlier, each reaction parameter (L‐tyrosine, CUS, Mg^2+^, ATP, CoA and malonate) was tested step wise. Here, the setup was assisted with a pick‐list generating script and an acoustic liquid handling robot, as described previously [33]. First, based on preliminary experiments, the concentration of the rate‐limiting enzyme, CUS, was set at 25 μM when investigating other reaction variables, while all other enzymes were set at 1 μM unless specified (Figure 2b). Next, BDMC biosynthesis was optimised through *Ec*CurA DiPro biosensor fluorescence, regarding ATP, Mg^2+^, CoA and malonate levels. Because of the non‐linear relationship of fluorescence to curcumin/BDMC concentration, we report relative fluorescence units (RFU). First, we found that the ATP level was critical to *Ec*CurA DiPro biosensor fluorescence with at least 1 mM required to reach saturation (∼65,000 RFU at endpoint—see Figure S5), while for optimal malonyl‐CoA supply, a concentration of greater than 2.5 mM malonate was required for maximal activity and fluorescence saturation. Here, both ATP and malonate are limiting substrates at low levels, but do not inhibit the reaction when in excess (Figure S5) In contrast, between 0.1 and 20 mM Mg^2+^, the fluorescence signal increase was linear up to 30 min of the reaction period. After this time point, biphasic activity was observed: less than 1 mM Mg^2+^ supported maximal activity and greater than 1 mM Mg^2+^ appeared to inhibit the formation of the reaction product and fluorescence (Figure 2b). A similar observation also occurred with the levels of CoA, with concentrations above or below the optimum CoA (0.25 mM) concentration. One potential interpretation of this response is a temporary depletion of free CoA availability (at less than 0.25 mM), thus leading to an imbalance between *p*‐coumaroyl‐CoA and malonyl‐CoA levels for the CUS enzyme. In contrast, if higher levels of CoA (>1 mM) were added, reduced *Ec*CurA DiPro biosensor fluorescence was observed (Figure 2b). Next, we used this optimised set of conditions to determine the optimal stoichiometry of enzymes in the biosynthetic pathway. To do this, each enzyme was varied in a 4‐fold dilution series from 0.0156 to 16 μM. First, some background fluorescence attributed to high levels of *p*‐coumaroyl‐CoA occurred if the level of the PCL enzyme was increased above 1 μM (Figure 2c and Figure S6). However, if the levels of the TAL and PCL enzymes were optimised to a peak concentration of 16 and 1 μM, respectively, maximal *Ec*CurA DiPro biosensor fluorescence (87,400 RFU) was reached, an 78‐fold increase from the starting condition when L‐tyrosine was limiting (50 μM). Interestingly, in experiments varying PCL and MatB together, a balance of these enzymes was required to prevent pathway inhibition, which suggests competition for free CoA (Figure 2c).

## 4 DISCUSSION

In summary, the curcumin DiPro biosensor permits rapid prototyping of a cell‐free biosynthetic pathway for a curcumin analogue in microscale conditions. While our experiments only provide a relative measure of pathway activity, they show that a finely tuned interplay between enzyme levels and the CoA cofactor is required for optimal pathway performance. This observation shows a clear advantage for studying enzyme ensembles using real‐time measurements in cell‐free conditions. In terms of biosensor output, fluorescent proteins, such as GFP, show a linear relationship between protein concentration and fluorescence. In contrast, similar to other ligand‐binding proteins, the fluorescence produced by the curcumin DiPro biosensor is controlled by binding kinetics, which are non‐linear. Examples of protein–ligand biosensors with non‐linear responses include Peredox [1] and Perceval [34]. These and similar examples show that is possible to use a sensor with non‐linear binding kinetics for studying reactions, provided that the linear range falls within the appropriate concentration window for the biological application. Here, we demonstrate one such application in the enzymatic synthesis of curcumin. While *Ec*CurA has a modest binding affinity for curcumin, it offers a strong fold‐change in the fluorescence signal upon binding (up to 15‐fold for curcumin—see Figure S3). While the substrate, curcumin, also has been shown to interact non‐specifically with amyloid fibres and fluoresce [28], the interaction with *Ec*CurA is stronger and more specific, leading to brighter fluorescence. In contrast to non‐specific interactions with proteins, we foresee *Ec*CurA and other DiPro biosensors as a customisable tool for a range of applications within synthetic biology that go beyond curcumin detection alone. An enzyme active site provides a distinct advantage for specific binding of target substrates, and the binding pocket can easily be engineered to detect other chemicals that interact and fluoresce upon binding. During our study, the highly related *Vv*CurA structure (PDB: 5ZXN) was released with NADP^+^ bound [30]. We compared *Vv*CurA to another related double bond reductase structure (PDB: 6EOW) with NADP^+^ and half‐curcumin (*p*‐hydroxybenzalacetone) bound [24]. Based on these structures, the predicted curcumin binding site of the *Ec*CurA DiPro biosensor is richly aromatic (Y53, Y64 and Y253), which may contribute to binding and the DiPro fluorescence mechanism, although further structural and biophysical characterisation is required to confirm this.

In conclusion, our report highlights a potential new DiPro biosensor mechanism using a fluorescent protein–substrate binary complex. This approach is advantageous as input to output signal propagation is rapid; it does not require any extra steps post gene expression or specific protein maturation requirements. We postulate that a wider variety of DiPro biosensors is available within literature and bioinformatics databases, particularly for pigmented small molecules with some level of intrinsic basal fluorescence—a required property for DiPro biosensors. A related literature example includes a range of computationally designed *α*‐helical coiled‐coil assemblies bound to synthetic hydrophobic dyes (e.g. 1,6‐diphenylhexatriene), structurally related to curcumin [35]. In addition, UnaG, a fluorescent bilin‐binding protein, was recently shown to bind and fluoresce with a range of synthetic chemical ligands [22]. Looking further, there are at least 300 known natural products, not including synthetic chemicals and analogues, with intrinsic fluorescence, which therefore provide a potential target for future DiPro biosensor development [36].

## CONFLICT OF INTEREST

The authors declare no conflict of interest.

## Supporting information

Supplementary Material S1

## Data Availability

All data is available from the corresponding author upon reasonable request.
