# Supplementary material for: A curcumin direct protein biosensor for cell‐free prototyping
Source: Eng Biol. 2022 Aug 18;6(2-3):62–8. doi: 10.1049/enb2.12024 (PMC9996706; doi:10.1049/enb2.12024)
Supplement: Supplementary file 1 — Supplementary Material S1 [file ENB2-6-62-s001.docx]

**Supporting information**

**
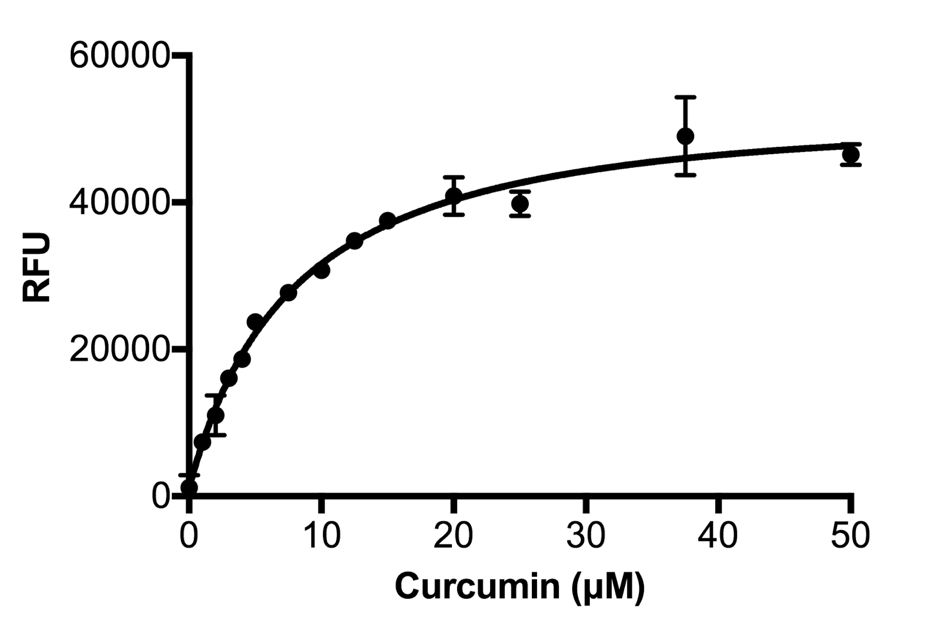
**

**Figure S1.** Fluorescence measurement of 25 μM *Ec*CurA with an increasing concentration of curcumin. Data is an average of two biological repeats of purified *Ec*CurA, prepared from separate batches.


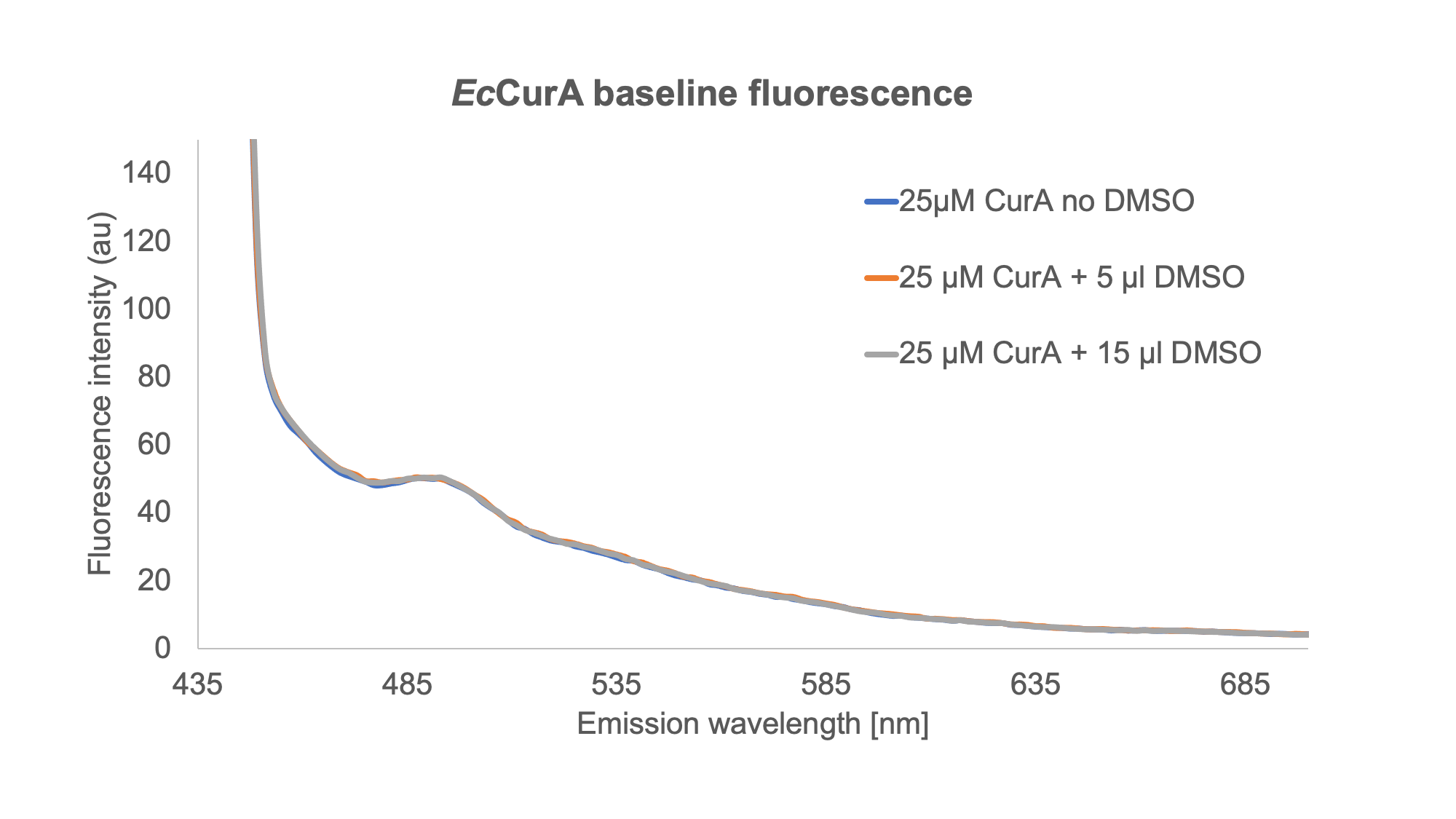


**Figure S2.** Baseline fluorescence of purified *Ec*CurA. Measurements were prepared in a 3 mL quartz cuvette. Excitation at 420 nm.


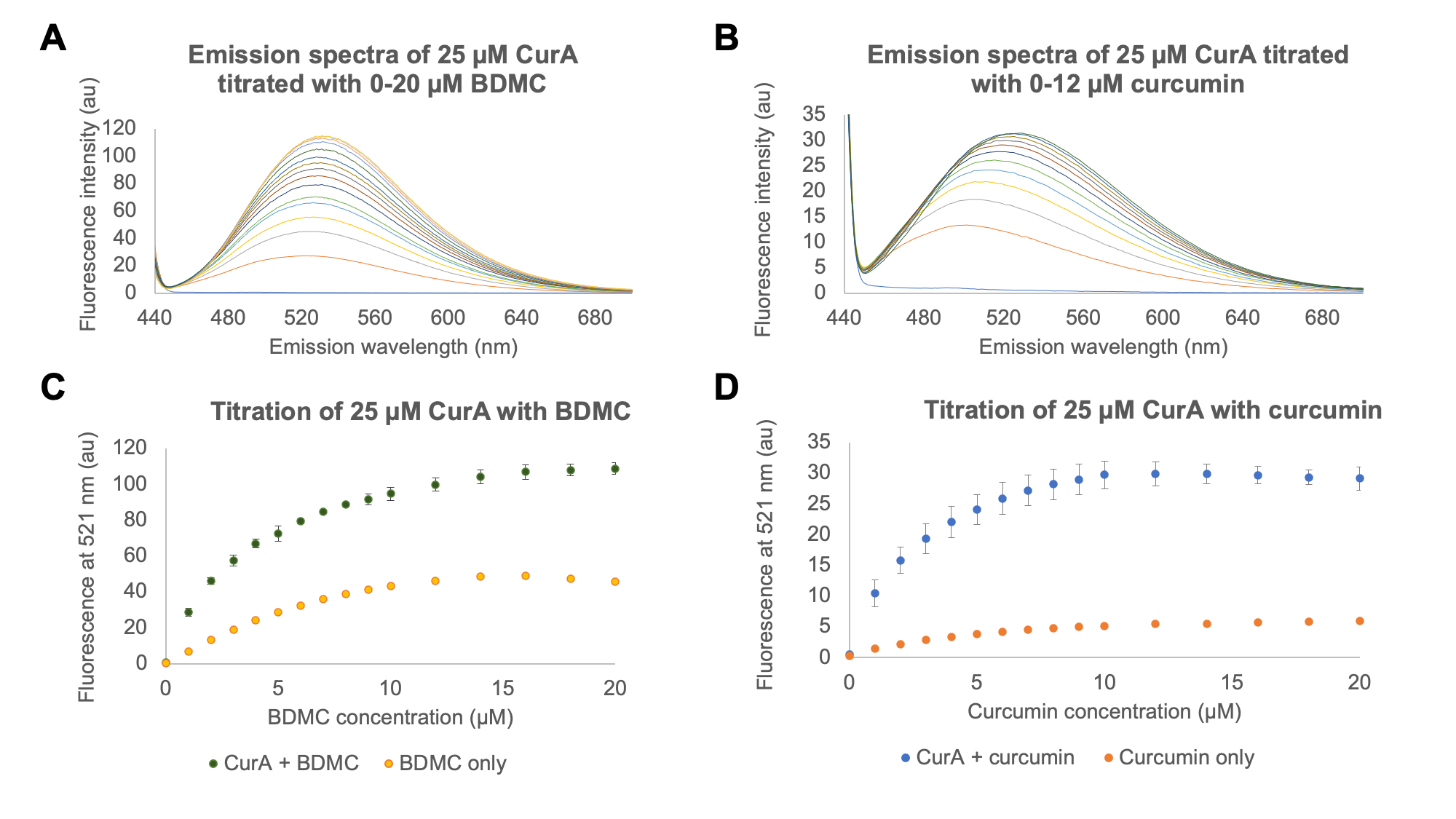


**Figure S3**. Fluorescence titrations of *Ec*CurA. (A) Emission spectra of *Ec*CurA-BDMC. (B) Emission spectra of *Ec*CurA-curcumin. (**C**) BDMC intrinsic fluorescence spectra in presence or absence of *Ec*CurA. (**D**) Curcumin intrinsic fluorescence spectra in presence or absence of *Ec*CurA. Measurements were prepared in a 3 mL quartz cuvette. Data is an average of two biological repeats of purified *Ec*CurA, prepared from separate batches. Excitation at 420 nm.

**
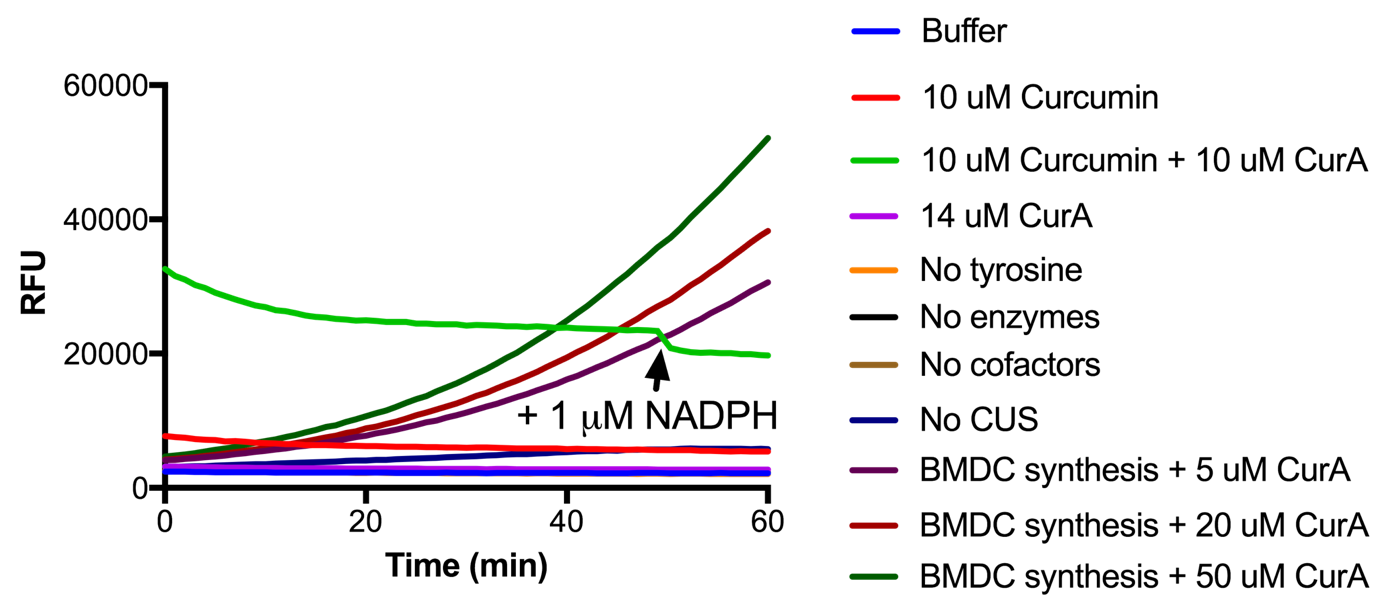
**

**Figure S4.** CurA time-course activity assay. An initial assay containing 1 mM L-tyrosine, 5 mM MgCl_2_, 10 mM malonate, 5 mM ATP, 0.25 mM CoA, 5 μM TAL, 5 μM PCL, 5 μM MatB, 5 μM CUS and 25 μM *Ec*CurA. Negative controls are shown whereby individual components of the reaction were omitted, thus showing relative background fluorescence. Unbound BDMC/curcumin has weak intrinsic fluorescence. Injection during the enzyme time-course reaction with 1 μM NADPH is shown by an arrow on the green trace (curcumin and *Ec*CurA). Data is an average of three technical repeats. These reactions are an average of triplicate technical repeat, with error bars removed for clarity.


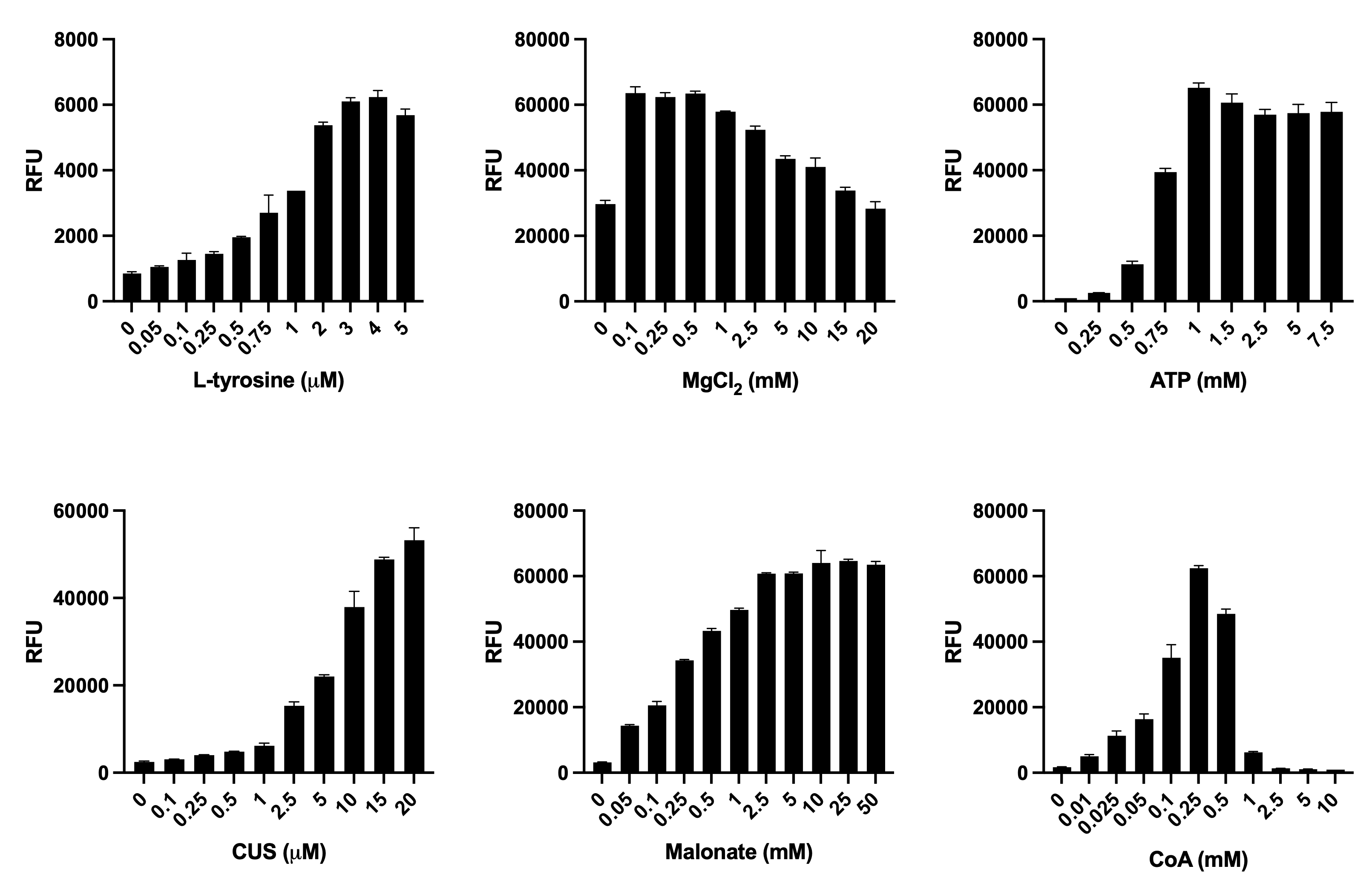


**Figure S5.** Endpoint (120 min) values from Figure 2B.


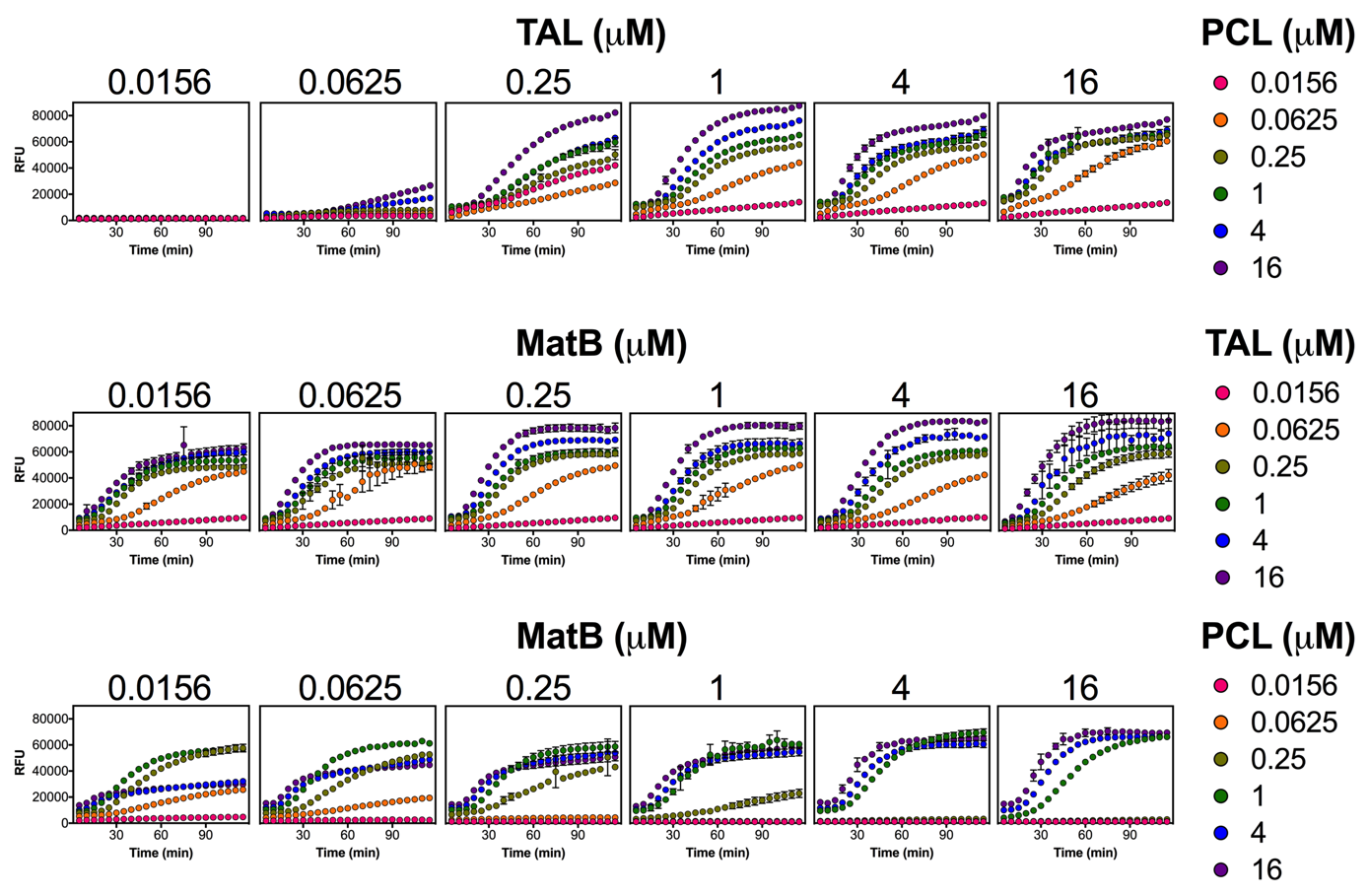


**Figure S6.** Optimisation of enzyme levels for the curcumin biosensor (Shown in **Figure 2C**). Enzyme levels were varied in a 4-fold dilution series from 16 to 0.0156 μM. Where two enzymes were varied, the third enzyme was kept constant at 1 μM. Conditions were Buffer A with 1 mM tyrosine, 0.25 mM CoA, 10 mM malonate, 1 mM MgCl_2_ and 5 mM ATP. 10 μL reactions were performed at 30°C as a technical triplicate repeat, prepared with the liquid handling robot. The assay was repeated as two biological repeats to ensure reproducibility.
